# Supplementary material for: The plant-specific transcription factors CBP60g and SARD1 are targeted by a Verticillium secretory protein VdSCP41 to modulate immunity
Source: eLife. 2018 May 14;7:e34902. doi: 10.7554/eLife.34902 (PMC5993538; doi:10.7554/eLife.34902)
Supplement: Supplementary file 2. — Sequences of primers used in this study. [file elife-34902-supp2.docx]

**Primers used in this study**

VdSCP41-knockout-up-F: 5’- GGGTTTAAUAGGGAGAATTGGTCGGAACT-3’

VdSCP41-knockout-up-R: 5’- GGACTTAAUGCAGAAGGACAAGGCTAGTGA-3’

VdSCP41-knockout-down-F: 5’- GGCATTAAUAGGCACCACATCATTTCC-3’

VdSCP41-knockout-down-R: 5’- GGTCTTAAUGACTTTGGTCTTCGTGCC-3’

VdSCP41-1300-F: 5’- ACAGGTACCATGAGGACCGAGACAG-3’

VdSCP41-1300-R: 5’- TCATTCGAACCAACCCATCCCGAG-3’

VdSCP41-FLAG-F: 5’- ACAGGTACCATGAGGACCGAGACAG-3’

VdSCP41-FLAG-R: 5’- TCATTCGAACCAACCCATCCCGAG-3’

VdSCP41- complement-F: 5’- AACCTCTAGAGGATCCGCCACCATGAGGACCGAGACAGCC-3’

VdSCP41- complement-R: 5’- GCAGCTTCTGCGAATTCCCAACCCATCCCGAGCAG-3’

VdSCP41N-FLAG-F: 5’- GACGAGCTCGGTACCATGAGGACCGAGACAGCCT-3’

VdSCP41N-FLAG-R: 5’- TTTGTAGTCTTCGAAGTTGCCGTTGAGGCTGTT-3’

VdSCP41C-FLAG-F: 5’- GACGAGCTCGGTACCATGCTTGCGACGCCAATCCTT-3’

VdSCP41C-FLAG-R: 5’- TTTGTAGTCTTCGAACCAACCCATCCCGAGCAG-3’

AtCBP60g-HA-F: 5’- CGAGGTACCATGAAGATTCGGAACAGCC-3’

AtCBP60g-HA-R: 5’- AGAGTCGACCAAGCCTTCCCTCGGATTTC-3’

AtSARD1-HA-F: 5’- ACAGGTACCATGGCAGGGAAGAGGTTA-3’

AtSARD1-HA-R: 5’- GGTCTCGAGGAAAGGGTTTATATGATTTTG-3’

GhCBP60b-HA-F: 5’- CGAGGTACCATGATGTTGCCTACTAAG-3’

GhCBP60b-HA-R: 5’- GCAGTCTACTGTACATATACCGTGTTG-3’

AtCBP60g-GFP-F: 5’- CGAGGTACCATGAAGATTCGGAACAGCC-3’

AtCBP60g-GFP-R: 5’- ACAACTAGTTTATTTGTATAGTTCATC-3’

Δ*sp*VdSCP41-mCherry-F: 5’- ACAGGTACCATGCTGACGCCAAATGATGTTC-3’

Δ*sp*VdSCP41-mCherry-R: 5’- TCATTCGAACCAACCCATCCCGAG-3’

Δ*sp*VdSCP41_-_*_nls_*-mCherry-F: 5’- ACAGGTACCATGCTGACGCCAAATGATGTTC-3’

Δ*sp*VdSCP41_-_*_nls_*-mCherry-R: 5’- TCATTCGAACCAACCCATCCCGAG-3’

VdSCP41-mCherry-F: 5’- ACATTCGAAATGGTGAGCAAGGGCGAG-3’

VdSCP41-mCherry-R: 5’- ACAACTAGTCTACTTGTACAGCTCGTC-3’

AtICS1-promoter-F: 5’-ACAGAATTCGTTTTACAACAGCAATG-3’

AtICS1-promoter-R: 5’-ACAGGTACCTGCAGAAATTCGTAAAGTG-3’

VdSCP41-NLuc-F: 5’- AGTGAGCTCATGAGGACCGAGACAGCC-3’

VdSCP41-NLuc-R: 5’- AGTCTCGAGCCAACCCATCCCGAGCAG-3’

GhCBP60b-VIGS-F: 5’-GCCGAATTCCCAATCTTGTCGCTCATC-3’

GhCBP60b-VIGS-R: 5’-GCACTCGAG ATGATGTTTCCAAGTAAGAG-3’

VdSCP41-RT-F: 5’- AACAGCCTCAACGGCAAC-3’

VdSCP41-RT-R: 5’- TTGGCGACGAGCGATTCC-3’

AtICS1-RT-F: 5’- TCCGTGACCTTGATCCTTTC-3’

AtICS1-RT-R: 5’- ACAGCGATCTTGCCATTAGG-3’

AtFMO1-RT-F: 5’- GGAGATATTCAGTGGCATGC-3’

AtFMO1-RT-R: 5’- TTTGGTTAGGCCTATCATGG-3’

AtCBP60g-RT-F: 5’- AAGAAGAATTGTCCGAGAGGAG-3’

AtCBP60g-RT-R: 5’- GGCGAGTTTATGAAGCACAG-3’

AtSARD1-RT-F: 5’- CCTCAACCAGCCCTACGTTA-3’

AtSARD1-RT-R: 5’- TAGTGGCTCGCAGCATATTG-3’

GhCBP60b-RT-F: 5’- TTTGGAACCTGACCCCAT-3’

GhCBP60b-RT-R: 5’- ATGATGTTGAGGATTGGTTC-3’

GhACTIN-RT-F: 5’-TCCCATTGAGCATGGGATCG-3’

GhACTIN-RT-R: 5’-CGTGAGAAGAACAGGGTGC-3’

VDAG_01962-His-F: 5’-ATGGGTCGCGGATCCGAATTCATGGACAGCTCATCGACTACTC-3’

VDAG_01962-His-R: 5’-TGGTGGTGCTCGAGTGCGGCCCTAGTTGGTCGGCGAGGTTCCCAGC-3’

VdSCP41_163-end_-F: 5'-GCCGCGCGGCAGCCATATGATCCTTGCGACGccaatc-3'

VdSCP41_163-end_-R: 5'-GTGGTGGTGGTGCTCGAGCCAACCCATCCCGAGCAG-3'

VdSCP41_101-163_-F: 5'-GCCGCGCGGCAGCCATATGCAAGCGGCCGATGTCATC-3'

VdSCP41_101-163_-R: 5'-GTGGTGGTGGTGCTCGAGggggaggttgggattgag-3'

EMSA-probe-F: 5'-TGTGTAATTTGGTGGTTATTTCATAGAAATTTTGGGGAAATTGTTGCACCTCTCCACTCT-3'

EMSA-probe-R: 5'-AGAGTGGAGAGGTGCAACAATTTCCCCAAAATTTCTATGAAATAACCACCAAATTACACA-3'
