# Supplementary material for: The plant-specific transcription factors CBP60g and SARD1 are targeted by a Verticillium secretory protein VdSCP41 to modulate immunity
Source: eLife. 2018 May 14;7:e34902. doi: 10.7554/eLife.34902 (PMC5993538; doi:10.7554/eLife.34902)
Supplement: Transparent reporting form [file elife-34902-transrepform.docx]

***eLife’s* transparent reporting form**

We encourage authors to provide detailed information *within their submission* to facilitate the interpretation and replication of experiments. Authors can upload supporting documentation to indicate the use of appropriate reporting guidelines for health-related research (see [EQUATOR Network](http://www.equator-network.org/%20)), life science research (see the [BioSharing Information Resource](https://biosharing.org/)), or the [ARRIVE guidelines](http://www.plosbiology.org/article/info:doi/10.1371/journal.pbio.1000412) for reporting work involving animal research. Where applicable, authors should refer to any relevant reporting standards documents in this form.

If you have any questions, please consult our Journal Policies and/or contact us: [editorial@elifesciences.org](mailto:editorial@elifesciences.org).

**Sample-size estimation**

- You should state whether an appropriate sample size was computed when the study was being designed
- You should state the statistical method of sample size computation and any required assumptions
- If no explicit power analysis was used, you should describe how you decided what sample (replicate) size (number) to use

Please outline where this information can be found within the submission (e.g., sections or figure legends), or explain why this information doesn’t apply to your submission:

We referred to previous publications from other groups and our group to choose an appropriate sample size.

For split luciferase imaging assay, we referred to the [methodological](http://dict.cn/methodological) publication (Chen et al., 2008, Plant Physiology) in which each data point consisted of at least three replicates and the student’s t test was performed to determine statistic significance of differences. In Figure 3C and Figure 3-figure supplement 3B, each data point consisted of at least 4 replicates generated from different leaves infiltrated with the same *Agrobacterium* strains.

For dual reporter analyses, we referred to our previous publications (Feng et al., 2012, Nature; Zhang et al., 2010, Cell host microbe) to include three replicates and use the student’s t test to determine statistic significance of differences. In Figure 4A, 4B, 5A and 5B, each data point consisted of three replicates generated by independent transformation.

For disease index analyses, we referred to our previous publications (Zhou et al., 2017, PLoS Pathogens; Zhang et al., 2017, New Phytologist) to include at least 12 plants for each inoculation in each experiment. Each data point consisted of three replicates generated from 24 *Arabidopsis* plants (8 plants for each replicate) or four replicates generated from 24 cotton plants (6 plants for each replicate) inoculated with the indicated strains.

For gene expression analyses, we referred to previous publications from other groups (Liu et al., 2013, PNAS) and our own (Zhang et al., 2010, Cell host microbe) to include three replicates from different plants or fungal cultures induced by different plants for analysis of statistic significance of differences. In Figure 2C, 2D, 7F, Figure 2-figure supplement 1D, Figure 2-figure supplement 2B and C, each data point consisted of three samples isolated from different plants. In Figure 1-figure supplement 1, each data point consisted of three samples isolated from fungal cultures induced by different plants. For SA measurement, each data point consisted of three samples induced with or without *V. dahliae* strains.

**Replicates**

- You should report how often each experiment was performed
- You should include a definition of biological versus technical replication
- The data obtained should be provided and sufficient information should be provided to indicate the number of independent biological and/or technical replicates
- If you encountered any outliers, you should describe how these were handled
- Criteria for exclusion/inclusion of data should be clearly stated
- High-throughput sequence data should be uploaded before submission, with a private link for reviewers provided (these are available from both GEO and ArrayExpress)

Please outline where this information can be found within the submission (e.g., sections or figure legends), or explain why this information doesn’t apply to your submission:

All of the experiments were repeated at least three times with similar results.

In the experiments of dual reporter analyses, different protoplasts independently isolated from different plants that are transformed with plasmids independently were considered as biological replicates (Figure legends in Figure 4A, 4B, 5A and 5B).

In the experiments of disease index analyses, different plants inoculated with different fungal cultures that are grown independently were considered as biological replicates (Figure legends in Figure 1E, 1F, 6 and 7E). Each data point in Figure 1F and 6 consisted of three replicates generated from 24 inoculated *Arabidopsis* plants (8 plants for each replicate). Each data point in Figure 1E and 7E consisted of four replicates generated from 24 inoculated cotton plants (6 plants for each replicate).

For gene expression analyses, different plants treated with peptides in multiple batch experiments were considered as biological replicates (Figure legends in Figure 2C, 2D, 7F and Figure 2-figure supplement 1D). Different batch fungal cultures induced by plants were considered as biological replicates (Figure legends in Figure 1-figure supplement 1).

For split luciferase imaging assay, different leaves infiltrated with *Agrobacterium* strains grown independently in multiple batch experiments were considered as biological replicates (Figure legends in Figure 3C and Figure 3-figure supplement 3B).

**Statistical reporting**

- Statistical analysis methods should be described and justified
- Raw data should be presented in figures whenever informative to do so (typically when N per group is less than 10)
- For each experiment, you should identify the statistical tests used, exact values of N, definitions of center, methods of multiple test correction, and dispersion and precision measures (e.g., mean, median, SD, SEM, confidence intervals; and, for the major substantive results, a measure of effect size (e.g., Pearson's r, Cohen's d)
- Report exact p-values wherever possible alongside the summary statistics and 95% confidence intervals. These should be reported for all key questions and not only when the p-value is less than 0.05.

Please outline where this information can be found within the submission (e.g., sections or figure legends), or explain why this information doesn’t apply to your submission:

The student’s t test was performed to determine statistic significance of differences and was illustrated in figure legends. The raw data and exact p values are list in the “source data” files.

(For large datasets, or papers with a very large number of statistical tests, you may upload a single table file with tests, Ns, etc., with reference to sections in the manuscript.)

**Group allocation**

- Indicate how samples were allocated into experimental groups (in the case of clinical studies, please specify allocation to treatment method); if randomization was used, please also state if restricted randomization was applied
- Indicate if masking was used during group allocation, data collection and/or data analysis

Please outline where this information can be found within the submission (e.g., sections or figure legends), or explain why this information doesn’t apply to your submission:

No group allocation was included in the study.

**Additional data files (“source data”)**

- We encourage you to upload relevant additional data files, such as numerical data that are represented as a graph in a figure, or as a summary table
- Where provided, these should be in the most useful format, and they can be uploaded as “Source data” files linked to a main figure or table
- Include model definition files including the full list of parameters used
- Include code used for data analysis (e.g., R, MatLab)
- Avoid stating that data files are “available upon request”

Please indicate the figures or tables for which source data files have been provided:

Source data for Figure 1E, 1F, 2C, 2D, 3C, 4A, 4B, 5A, 5B, 6, 7E, 7F, Figure 1-figure supplement 1, Figure 2-figure supplement 1D, Figure 2-figure supplement 2A, B, C and Figure 3-figure supplement 3B have been provided in “Source data” files.
